# Reconsidering the lives of the earliest Puerto Ricans: Mortuary Archaeology and bioarchaeology of the Ortiz site

**DOI:** 10.1371/journal.pone.0284291

**Published:** 2023-04-26

**Authors:** William J. Pestle, Elizabeth M. Perez, Daniel Koski-Karell

**Affiliations:** 1 Department of Anthropology, University of Miami, Coral Gables, FL, United States of America; 2 National Institute of Archaeology, Washington, D.C., United States of America; University of Bern: Universitat Bern, SWITZERLAND

## Abstract

We possess rather little detailed information on the lives of the first inhabitants of Puerto Rico—the so-called “Archaic” or “Pre-Arawak” people—despite more than a century of archeological research. This is particularly true bioarchaeologically, as fewer than twenty burials of the several millennia of the Archaic Age have been recovered, let alone analyzed in any detail. Here, we present the results of archeological, osteological, radiometric, and isotopic analysis of five individuals from the Ortiz site in Cabo Rojo, southwestern Puerto Rico. Study of these previously unpublished remains, which represent a 20–25% increase in the sample size of remains attributed to the period, provides many critical insights into earliest Puerto Rican lifeways, including aspects of mortuary practice, paleodiet, and possibly even social organization. A review of their burial treatment finds a mostly standardized set of mortuary practices, a noteworthy finding given the site’s potential millennium-long use as a mortuary space and the possibly distinct place(s) of origin of the individuals interred there. Although osteological analysis was limited by poor preservation, we were able to reconstruct aspects of the demography that indicate the presence of both male and female adults. Stable isotope analysis revealed dietary differences from later Ceramic Age individuals, while dental pathology indicated heavy masticatory wear attributable to diet and/or non-masticatory function. Perhaps most crucially, direct AMS dating of the remains confirms these as the oldest burials yet recovered from the island, providing us both with a glimpse into the lives of some of the island’s first inhabitants, and with tantalizing clues to the existence of a different degree of cultural “complexity” than is often ascribed to these earliest peoples. The existence of what radiocarbon dates suggest may be a persistent formal cemetery space at the Ortiz site has potentially significant implications concerning the territoriality, mobility, and social organization of the earliest peoples of southwestern Puerto Rico.

## Introduction

Although the lifeways of Puerto Rico’s earliest inhabitants have been investigated for over a century, they remain poorly understood because of both methodological shortcomings and an interpretive framework that privileged later, Ceramic Age, cultural manifestations and promoted caricatured views of earlier groups [1]. Knowledge of the bioarchaeology and mortuary practices of the island’s earliest inhabitants is limited further due to the paucity of well-preserved, scientifically excavated, and thoroughly analyzed human burials. Fewer than twenty individuals belonging to this approximately 4000-year-long period have been identified, a small subset of which has been analyzed employing anything approximating current methods.

In this work, we present detailed reconstructions of the lives of five heretofore unpublished individuals from the Ortiz site in southwestern Puerto Rico. In doing so, we address a series of specific questions about mortuary practice, taphonomy, biological profile, chronology, paleodiet, and mobility. Our findings are based on a combined program of archaeological, taphonomic, osteological, radiometric, and stable and radiogenic isotopic analysis. Ultimately, our efforts determined that: 1) the Ortiz burials are the oldest directly dated individuals from the island of Puerto Rico, 2) that there was a common canon of mortuary treatment despite the burials having been made over a period of five to ten centuries, 3) that their dietary practices were notably different than that of other contemporary and later populations, and 4) that, based on paleomobility data, the Ortiz site likely served as a formal central burial precinct for individuals (male and female) from nearby localities.

While the results of this study are contingent due to the poor condition of the remains and limited available excavation documentation, this work nonetheless contributes significantly to our understanding of lived experiences and community mortuary practices of a group of Puerto Rico’s early inhabitants. This analysis also provides insights into broader issues presently animating investigation of the island’s first inhabitation, including concerns surrounding settlement stability, site persistence, and notions of territoriality.

### Culture history

The pre-Columbian culture history of Puerto Rico has been regarded as consisting of three quasi-distinct Ages (Archaic, Ceramic, and Historic), as defined by Irving Rouse beginning in the 1940s [1]. While the Archaic Age was longest lasting of these three, it remains the least well understood.

In our work [e.g. 2], we have chosen to follow researchers such as Rivera-Collazo [3] and to reject the term “Archaic” because it has a pejorative connotation, is historically inaccurate, and homogenizes 4,000 to 5,000 years of human cultures across the vast insular Caribbean. We also decline to use the term “Pre-Arawak” [4] for the region’s early inhabitants, as it identifies such groups by what they were not and by whom they may have been succeeded. We believe it is more appropriate to consider time and space in naming these people, and here refer to the subjects of this research as “the earliest inhabitants of Puerto Rico”.

The initial arrival of humans in Puerto Rico appears to have occurred by not later than the middle third millennium BC. Recent Bayesian modeling indicates that human colonization began between 4670 to 4310 calibrated radiocarbon years before present (cal rcybp), equivalent to 2720 to 2360 cal B.C. at 95.4% confidence [5]. Genomic analyses [6, 7] have posited one or two migratory waves for these early inhabitants of the insular Caribbean. However, as these genetic reconstructions of biological origin do not include individuals from Puerto Rico, we are not certain whether they can be generalized to include it.

Puerto Rican archaeological sites putatively associated with early occupation most often are small (< 500 m^2^) shell middens located near present-day coastlines. Such sites commonly include large concentrations of marine shell along with smaller quantities of lithic artifacts, vertebrate fauna, and floral remains [1, 8, 9]. Investigation of most middens do not report the presence of subsistence remains from all locally available resources, which has been interpreted as evidence for residential mobility and food-foraging [8–11]. Numerous lithic workshop sites of the period also have been found at or near natural deposits of preferred raw materials such as chert [12–16].

A small number of larger and spatially complex sites containing substantial artifact variety provide evidence of greater diversity in behavioral activity than the smaller and simpler exploitation and workshop sites might suggest. Such sites include Angostura [3, 17–19], Cueva María de la Cruz [20, 21], Maruca [22–24], and Puerto Ferro [25–28]. These sites contain human burials, artifacts and raw materials of non-local origin, and overall contents that evince a wide range of domestic and manufacturing activities. Furthermore, pottery fragments encountered at certain early sites demonstrate the use of ceramics by at least some early groups [4]. These characteristics do not comport fully with a notion that the island’s earliest inhabitants were itinerant, acephalous, food foragers.

### Regional and site setting

Southwestern Puerto Rico, especially the *municipio* of Cabo Rojo, is the location of an abundance of archaeological sites belonging to the island’s early habitation period. Site files held by the State Historic Preservation Office/*Oficina Estatal de Conservación Histórica* (SHPO/OECH) and the *Consejo de Arqueología Terrestre*/Council of Terrestrial Archaeology of the *Instituto de Cultura Puertorriqueña* (ICP) show that over 60% of documented sites in the region are associated with these early inhabitants. This is very different from site frequencies for pre-Columbian sites found elsewhere on the island. Why the southwest region contains this usually large proportion of early sites is unclear.

**Fig 1:**
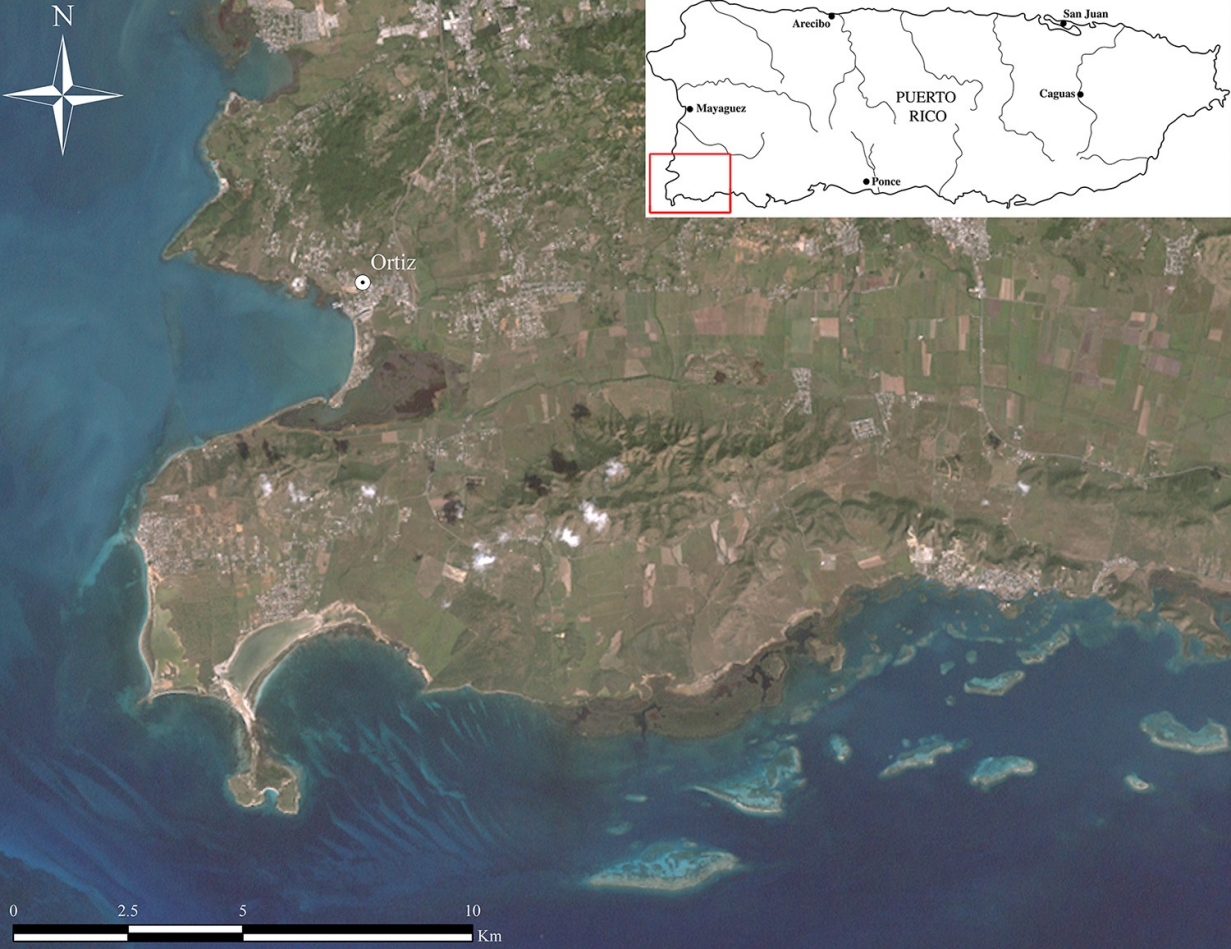
Location of Ortiz site. Inset map of Puerto Rico with area of main figure indicated by red box. Satellite imagery from LANDSAT GLS, inset map drawn by Jill Seagard.

The Ortiz site (ICP catalog number CR-88-03-02; 18.03° N, 67.17° W) is in the *Boquerón barrio* of Cabo Rojo (Fig 1). It is situated atop a prominent hill (25 m) overlooking the sheltered waters of Boquerón Bay. In the mid-1980s, the parcel where the Ortiz site is located was proposed for residential development. Compliance with Puerto Rican cultural resource management (CRM) requirements led to a 1987 archaeological survey, which discovered the site and found it to be potentially significant [29]. That preliminary finding was confirmed in a subsequent (1993) evaluation investigation [30], which identified the site’s boundaries, and excavated four 2 x 2 m test units under direction of one of the authors (DKK).

The 1993 excavations revealed a roughly 500 m^2^ site that included a 30–40 cm deep midden deposit consisting of abundant marine invertebrate remains [2], copious lithic artifacts representing all stages of stone tool manufacture, and lesser quantities of vertebrate fauna remains, coral, and other artifacts. These excavations also encountered five human burials, the subject of the present manuscript. Post-excavation analysis and reporting of these findings was not completed, and the excavation director subsequently curated the Ortiz site collection until its transfer in 2019 to the University of Miami, where the materials are under study before being returned to Puerto Rico.

We interpret the Ortiz site to have been a habitation location during at least some portion(s) of its apparent multi-millennial existence. This is based on its location, abundant subsistence remains, variety of recovered artifacts including products and debitage apparently representing all stages of lithic tool manufacture, and the presence of human burials. The recovery of multiple burials associated with Puerto Rico’s earliest habitation period places the Ortiz site on a very short list of early mortuary spaces.

### Comparanda

**Fig 2:**
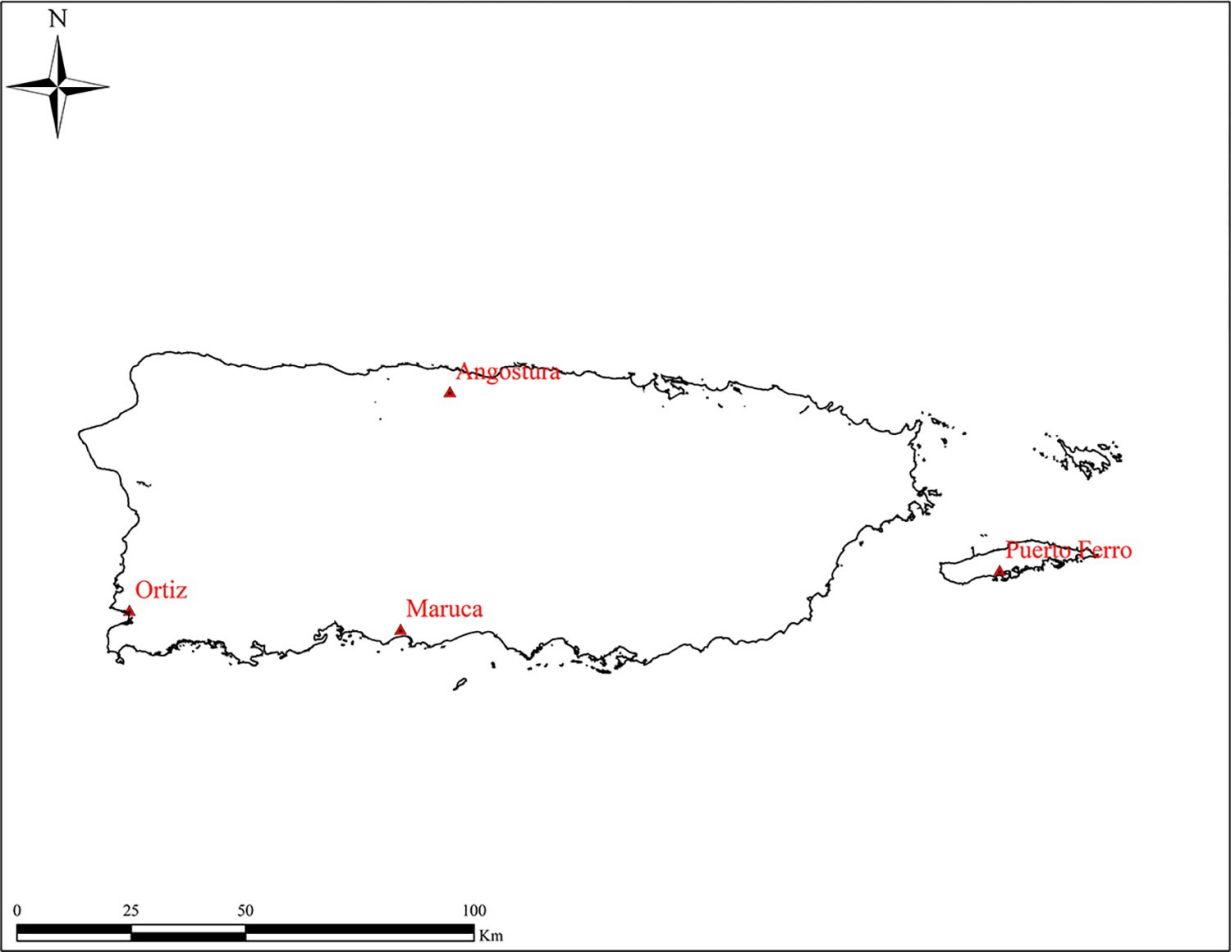
Map indicating location of Puerto Rican sites with purportedly early period (“Archaic”) burials. Map derived from *Junta de Planificación de Puerto Rico* y and 2012 U.S. Census.

Human burials associated with Puerto Rico’s early occupation period have been recovered from five sites on the main island and the adjacent island of Vieques (Fig 2). Useful radiocarbon dates, osteological, or isotopic data are available for only a subset of this already small sample. While information from other previously analyzed early remains is limited, it is nonetheless useful for comparison with data obtained from the Ortiz site skeletons.

Excavators of the Angostura Site on Puerto Rico’s north-central coast encountered a putatively early primary burial and several secondary burials [17]. Charcoal samples associated with the primary burial yielded radiocarbon dates of 3570±40 and 3670±40 rcybp (circa 1530 to 1710 BC). The Angostura human remains have not been analyzed. The Cueva María de la Cruz Site is located in the island’s northeast. Its excavation reports describe the presence of as many as three fragmented or partial, and possibly secondary, burials in the site’s deepest and earliest levels [20, 21]. These burials were not radiocarbon dated nor were the remains analyzed. Investigation of the Puerto Ferro Site on the island of Vieques recovered one burial, which was given the name “El Hombre de Puerto Ferro” [25, 26]. While no direct date was obtained for this individual, the Puerto Ferro Site had been dated to around 1900 BC. These skeletal remains have undergone detailed osteological analysis [28].

The most useful data for comparison with the Ortiz Site human remains comes from the site of Maruca in Puerto Rico’s south-central city of Ponce. Excavations at Maruca yielded 11 human burials [22–24]. Although those skeletons have not been dated directly, the site has produced eight radiocarbon dates ranging between 4840–2445 rcybp (circa 1840 to 445 BC). This confirms the Maruca Site’s association with the island’s first period of inhabitation. The 11 Maruca individuals have undergone thorough mortuary and osteological analysis [31], and stable isotope data are available for three of them [32]. This makes Maruca particularly important for comparison with our analysis of the Ortiz remains.

In addition, there are a far larger number of individuals (more than 200) dating to the later Ceramic Age of Puerto Rico that have been the subject of osteological, radiometric, and isotopic study by Crespo Torres and one of this study’s authors, WJP [33–36]. Comparison of these later individuals with the (presumably) earlier burials from Ortiz may shed light on similarities and differences between these two purportedly distinct phases of the island’s culture history.

## Research questions

The present work was intended to answer the following seven research questions:

1. What were the funerary traditions of the earliest populations of southwestern Puerto Rico?
2. How have taphonomic processes affected the preservation/condition of the Ortiz individuals?
3. What is the biological profile (age-at-death, sex, and stature) of the Ortiz sample?
4. Using direct dating of the osteological remains, what is the chronology of the Ortiz burials?
5. What were the paleodietary practices of the Ortiz individuals, as based on stable isotope analysis and multi-source mixture modeling?
6. Based on analysis of Sr isotopes, were the Ortiz individuals of local or non-local origin?
7. What broader conclusions about the lifeways of the early populations of southwestern Puerto Rico can be gleaned from the answers to the questions enumerated above?

While our findings remain contingent due to the poor condition of the remains and the limited available excavation documentation, this work nonetheless contributes to understanding lived experiences and cultural practices of a group of Puerto Rico’s early inhabitants.

## Materials and methods

Our understanding of the bioarchaeology of Puerto Rico’s first people is limited by at least two factors: poor preservation and small sample size. Recovered human remains of the island’s earliest inhabitants total fewer than two dozen individuals, and they are generally in very poor condition due to thousands of years of exposure to the neo-tropical environment [37]. The Ortiz site individuals are no exception, as all five individuals are in very poor condition. Given the circumstances, we are unable to follow a traditional bioarchaeological approach, which derives statistical inferences from large samples sizes. Instead, we employ a more fine-grained approach seeking to extract the greatest amount of information possible from a small and contingent study sample.

The Ortiz Site individuals were designated in the order encountered during field excavation. They consist of Burial 1 (B1), Burial 2 (B2), Burial 3 (B3), Burial 4 (B4), and Burial 5 (B5). These remains presently are located at the University of Miami (Coral Gables, Florida, USA), before their return to Puerto Rico. All necessary permits were obtained for the described study (specifically, permission for analysis granted by the *Consejo para la Protección del Patrimonio Arqueológico Terrestre*, *Instituto de Cultura Puertorriqueña*), which complied with all relevant regulations.

### Burial practices and funerary taphonomy

Examination of the Ortiz burial remains began by assessing their taphonomic condition and consideration of their archaeological context. Taphonomy was assessed through documentation of coloration, consistency, and degree of erosion and weathering of all available elements following the guidelines of Buikstra and Ubelaker [38]. The position of each skeleton’s bones when excavated was documented in field notes, photographs, and drawings. This allowed us to postulate as to a body’s positioning when buried, spatial arrangement of the bodies in relation to one-another, any post-depositional movement of the remains, and temporal relationships among the five burials. Our assessment of burial treatment and positioning followed methods outlined in Knüsel and Robb [39:Table 1]. In particular, we were guided by their differentiation among:

1. “Primary Depositions”, which reflect, “(t)he original placement of the corpse […] inferred when bones are in anatomical articulation, modified only by the processes of decomposition in situ,”
2. “Secondary Depositions,” which reflect (a) subsequent placement of human remains, following movement from their primary location; often inferred when persistent articulations are disarticulated,” and,
3. “Collective Deposition,” which are, “human remains deposited successively over time rather than in a single episode.”

**Table 1:** Food group isotope and macronutrient composition used in the FRUITS modeling of paleodiet.

		Macronutrient concentration (%)		Tissue *δ*^13^C (‰)			Tissue *δ*^15^N (‰)
Food grouping	Group *n = 611*	Protein	Energy	Bulk	Protein	Energy	Protein
C_3_ plants	67	14±12	86±14	-25.5±1.7	-26.5±1.7	-25.3±1.7	3.3±2.1
C_4_/CAM plants	20	14±12	86±14	-10.4±1.3	-11.4±1.3	-10.2±1.3	5.1±2.9
Freshwater/terrestrial fauna	115	73±14	27±15	-19.8±2.9	-17.6±2.9	-25.8±2.9	6.2±2.7
Marine 1 (M1)	131	76±15	24±17	-13.9±2.1	-12.7±2.1	-17.8±2.1	13.0±2.7
Marine 2 (M2)	278	76±15	24±17	-10.7±3.3	-9.5±3.3	-14.6±3.3	5.9±3.0

### Osteological examination

Osteological analysis, as preservation and completeness allowed, consisted of the following: 1) inventory of skeletal and dental remains, 2) metric data collection, 3) determination of age and sex, 4) estimation of living stature, and 5) documentation of pathological or traumatic conditions.

This analysis began with inventory of each individual burial and its archaeological context. The presence, completeness, and siding of each identifiable skeletal element were assessed using lists of all possible skeletal elements and standard anatomical drawings. We next recorded measurements of the available skeletal and dental elements using sliding and spreading calipers and a flexible tape. The landmarks and definitions of measurements and indices employed were derived from Bass [40], Buikstra and Ubelaker [38], and White and Folkens [41].

Assessment of biological sex focused on known sexually dimorphic cranial and pelvic traits, a suite of postcranial metrics, and cranial, dental, and post-cranial discriminant functions. Examination for morphological features of the bony pelvis and skull was guided by reference to Bass [40], Buikstra and Ubelaker [38], and White and Folkens [41], and Schwartz [42]. Metric indices, when applicable, were derived from Bass [40] and discriminant functions from Ditch and Rose [43], and Giles [44]. As all five Ortiz Site individuals were adults; age-at-death was assessed by the state of the pubic symphysis [45, 46], auricular surface [47, 48], cranial sutures closure [38, 49], and dental wear [50].

Living stature for these individuals was reconstructed based on the excavation’s in-situ measurements. While we acknowledge the potential shortcomings of this approach, the state of osteological preservation did not allow for extrapolation of stature from long bone lengths. As the burials were primary in nature (see below), we remain confident in the accuracy of the in-situ measurements of stature.

Assessment and differential diagnosis of pathological and traumatic changes in the skeletal material was made by reference to standard images and descriptions presented in Aufderheide and Rodriquez-Martin [51], Steckel and Rose [52], and Ortner [53, 54], with documentation of trauma guided by the works of Merbs [55] and Ornter [53, 54], and Walker [56, 57].

### Radiocarbon dating

Direct (bone collagen) AMS dating of all five Ortiz individuals was conducted at Beta Analytic, Inc. in Miami, Florida. Their AMS procedures are detailed at <https://www.radiocarbon.com/beta-lab.htm>. Collagen samples for radiocarbon dating were obtained through two differing protocols. Collagen extraction for Burials 1, 2, and 4 was performed at Beta Analytic following that laboratory’s in-house protocols (<https://www.radiocarbon.com/pretreatment-carbon-dating.htm>). WJP extracted collagen from Burial 3 using a modified Longin [58] method as described in Pestle [36]. No collagen could be extracted from Burial 5 despite repeated attempts.

AMS dates were calibrated using OxCal 4.4 [59] and a mixed curve of Intcal20 [60] and Marine20 [61] with marine carbon percentage derived from paleodietary modeling of each individual (described below) and a local, Marine20 adjusted, ΔR of -138 ± 23 [62]. The results for each burial are presented by reference to their two-sigma range (95.4% confidence) calibrated ages. There are difficulties inherent in the direct dating of ancient bone from tropical coastal environments, which include the potential for poor collagen preservation and the possible inclusion of ^14^C-depleted marine carbon. Despite that, we believed this direct dating approach would provide the most accurate assessment of burial chronology at the site.

### Paleodiet and stable isotope analysis

Following extraction (detailed above), bone collagen was analyzed by IRMS at Beta Analytic to generate *δ*^13^C_co_ and *δ*^15^N_co_ values (https://www.radiocarbon.com/dietary-isotopic-analysis.htm).

Sample preservation quality was determined using both chemical (collagen and hydroxyapatite yield) and elemental (carbon and nitrogen yield, atomic C/N ratio) data. Only well-preserved samples (collagen yield >0.5 wt%, carbon yield >4.5 wt%, nitrogen yield >0.9 wt%, atomic C/N ratio between 2.9–3.6) were included in subsequent paleodietary modeling. Hydroxyapatite extraction followed protocols first established in Lee-Thorp [63] and Krueger [64] and modified by Pestle [36]. Hydroxyapatite isotopic analysis was performed in the Marine Geology and Geophysics Stable Isotope Laboratory at the Rosenstiel School of Marine and Atmospheric Science, University of Miami. Samples were analyzed using a Kiel-IV Carbonate Device coupled to a Thermo Finnigan DeltaPlus IRMA, providing *δ*^13^C_ap_ values. Results were calibrated using an OCC (optically clear calcite) standard calibrated to NBS-19.

Individual isotopic data were subsequently analyzed using the Bayesian multi-source mixture model FRUITS v2.1.1 (Food Reconstruction Using Isotopic Transferred Signals) [65]. This allows for probabilistic and uncertainty-integrated quantification of dietary inputs. We chose the FRUITS program because of its ability to incorporate food macronutrient, elemental, and isotopic composition data, along with source and consumer uncertainty, in its calculations. The modeling parameters employed here are identical to those used in Pestle, et al. [66] for modeling the diets of 229 Ceramic Age individuals from Puerto Rico, allowing for direct dietary comparison between these two different cultural groups. Comparison can also be made with the Maruca Site burial designated 2B [32], whose diet was modeled using the same methods. All FRUITS simulations for the Ortiz site were performed using 10,000 iterations, as recommended by the software’s developers.

Prior to FRUITS modeling, human isotope data were converted to account for fractionation with the offset for *δ*^13^C_ap_ stipulated as 10.1±0.4‰ following Fernandes and colleagues [67]. For *δ*^15^N_co_, we employed a trophic fractionation value of 3.6±1.2‰ as recommended from experimental studies of omnivorous animals [68–73]. Finally, the consumer-foodstuff offset and error for *δ*^13^C_co_ was calculated using the linear regression method described in Pestle and colleagues [74]. Instrumental uncertainties of 0.1‰ were additionally stipulated for all isotope systems. Elemental routing was specified as follows: all nitrogen in bone collagen was stipulated as coming from dietary protein, carbon in hydroxyapatite was stipulated to be a weighted average of all dietary carbon, and the carbon in bone collagen was stipulated as approximating a 3:1 ratio (74±4%:26%) of dietary protein to energy [67].

Reference to a variety of previous studies provided isotopic data on 611 potential faunal and floral foodstuffs [36, 75–91]. All modern data had their *δ*^13^C values corrected by +1.5‰ to account for the Suess/fossil fuel burning effect [92, 93]. Additional corrections concerning differences between measured and edible tissues are detailed in Pestle [36:Table 38].

The FRUITS model requires the *a priori* stipulation of food groups/sources to be used in mixture modeling (Table 1). Two flora groups were designated (C_3_ and C_4_/CAM plants) using the well-established basis of isotopic differences in plants’ photosynthetic pathways. Food group determination was more complex for fauna, and included consideration of isotopic and macronutrient chemical composition, ecological niche, and trophic level [94]. Due to isotopic and macronutrient similarities, we determined that freshwater and terrestrial (FWTERR) fauna would be combined into one group for use in subsequent modeling.

Conversely, due to isotopic differences among the marine taxa analyzed, two distinct marine food groupings were stipulated. The group designated Marine 1 (M1) consists primarily of higher trophic level and/or pelagic taxa having higher *δ*^15^N and lower *δ*^13^C values. The group designated Marine 2 (M2) is composed of shallow water, reef, and seagrass taxa having lower *δ*^15^N and higher *δ*^13^C signatures. Group M2 includes shellfish that live in shallow water, a significant subsistence resource for the Ortiz site inhabitants, as evidenced by the abundant marine shell in the site’s midden.

Macronutrient composition for each of these food groups was determined by reference to the United States Department of Agriculture (USDA) National Nutrient Database for Standard Reference [95]. Elemental composition, particularly %C, of each foodstuff/macronutrient group was based on formulae provided in Morrison et al. [96]. Digestibility was determined following Hopkins [97].

Carbon isotope offsets between measured bulk food isotope values and the isotopic values of a foodstuff’s fats (bulk-6‰) and carbohydrates (bulk+0.5‰) were based on data from Tieszen [98]. The carbon isotope signature of a measured foodstuff’s protein was determined using a mass-balance equation. This was done so that a weighted average of the *δ*^13^C of protein and energy (fats and carbohydrates) would equal the measured *δ*^13^C bulk value as corrected for the concentration of carbon in each macronutrient and foodstuff-appropriate macronutrient concentration. Table 1 presents the final food group isotope and macronutrient composition as used in the FRUITS simulations.

To facilitate direct comparison among and between the diets of different individuals and groups (Ceramic Age, Maruca, and Ortiz), we performed principal component analysis (PCA) of the foodstuff contribution data produced using FRUITS. This process linearly transformed the five dimensions of the FRUITS data into two principal components, which together accounted for 89.2% of the variance observed in the sample. PCA was conducted in R [99] using the FactoMineR [100] and factoextra [101] packages.

### Strontium isotope analysis

Strontium (Sr) isotope analysis of early-forming teeth can provide useful information concerning where persons were born or spent the earliest years of their life. Sr isotope ratios vary geographically based on underlying geological differences [102, 103], as well as due to differing atmospheric and marine effects [104, 105]. Sr atoms can replace calcium in the crystalline structure of hydroxyapatite [102], the mineral that forms the preponderance of dental enamel [41]. As this tissue does not undergo metabolic turnover *in vivo* and preserves quite well in the burial environment [106], it forms an ideal proxy for the geochemical environment, and location of growth, for an individual’s early forming teeth [102, 107]. This analysis thus was used to assess the possible place of origin of four Ortiz Site individuals, as has become ubiquitous in bioarchaeology.

The buccal surfaces of molar crowns from Burials 1, 3, 4, and 5 were cleaned by means of abrasion using a stainless-steel rotary tool bit. A fresh bit was used to remove a transect of ground enamel weighing 5–10 mg. Samples were cleaned by multiple rounds of sonication and rinsing with MilliQ water (18 mΩ). Rinsed samples were dried in an oven at 40°C for 24 hours and then homogenized using an agate mortar and pestle. Diagenetic contaminants were removed by sequential treatment with 0.1 N acetic acid using a ratio of 1.0 ml of acid for every 20 mg of powder following protocol number two of Hoppe et al. [108]. Five mg of homogenized sample was dissolved in 5 mL of 0.25 M HNO_3_ to minimize leaching of strontium from detrital/clay residues. The solution’s molarity was subsequently adjusted to 6 M by adding 4.5 mL of concentrated HNO_3_. Low-blank separation of strontium from each sample was achieved by extraction chromatography using Eichrom Sr resin following the methods of Pourmand et al. [109], and Pourmand and Dauphas [110]. In doing this, dissolved samples were introduced to the columns in 6 M HNO_3_ at the rate of 1 mL per minute. Columns were rinsed with 20 mL of 6 M HNO_3_ to remove the matrix elements. Finally, Sr was eluted from the columns with 10 ml of 0.01 M HNO_3_.

High precision strontium isotope analysis was performed on a Thermo Fisher Neptune Plus^TM^ MC-ICP-MS and a quartz stable sample introduction (SSI) system at the Neptune Isotope Laboratory (NIL), Rosenstiel School of Marine and Atmospheric Science, University of Miami. The measured ^87^Sr/^86^Sr ratios were corrected for mass bias and isobaric interferences and the final ratio was further adjusted relative to the accepted value of 0.710248±0.000003 for SRM 987 [111] to allow comparison with literature measurements of radiogenic Sr isotopes. Every 1–5 sample measurements were bracketed with measurements of two SRM 987 standard solutions at 100 ng g^-1^. The strontium isotope measurement uncertainty of NIST SRM 987 standard solution was ±0.000007 (%95 CI) for ^87^Sr/^86^Sr. Results were compared with bioavailable Sr isotope data for Puerto Rico [112–114] for identification of possible individual place of origin.

## Results

### Burial practices and funerary taphonomy

**Fig 3:**
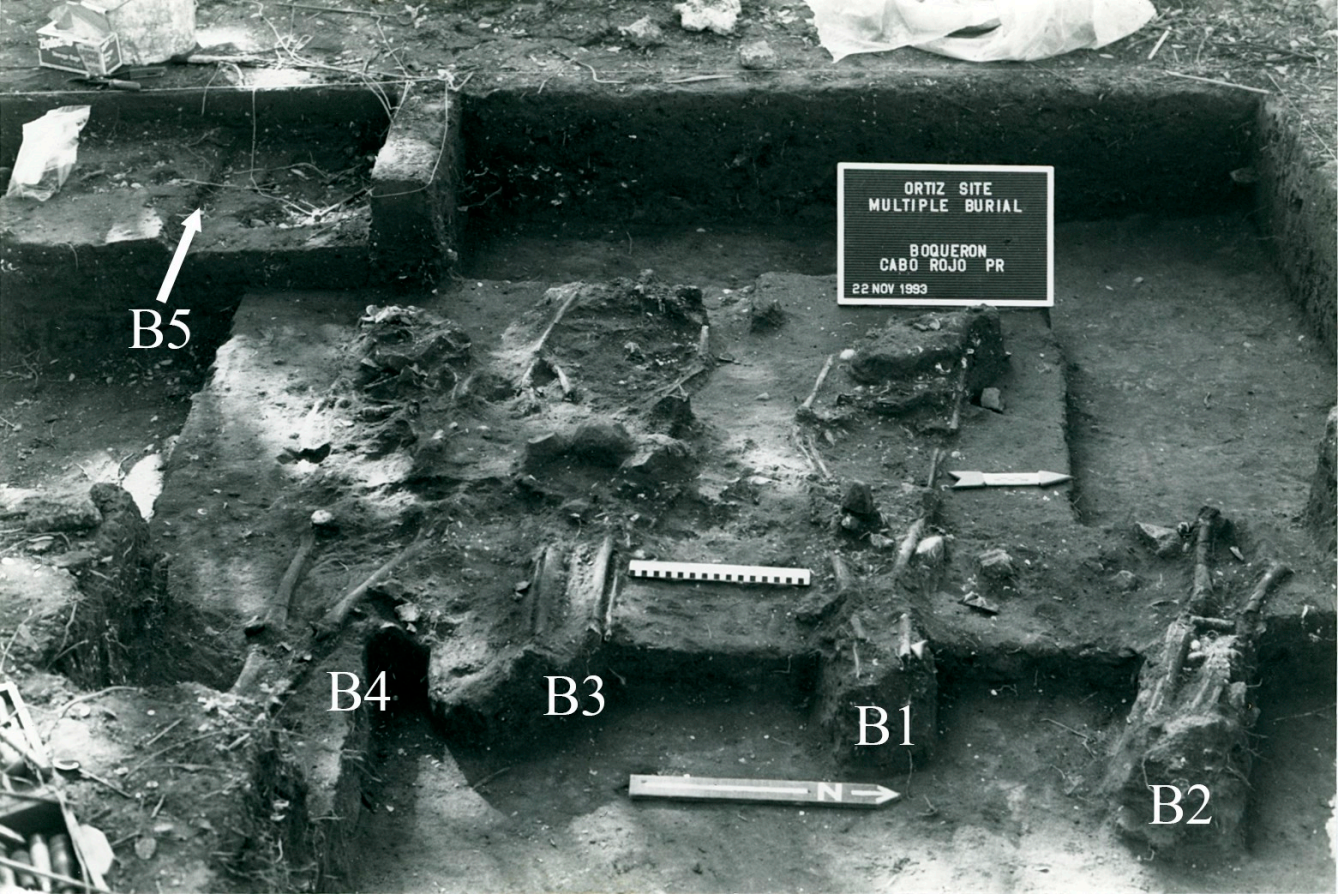
November 1993 photograph of Ortiz burials in situ.

The five inhumations excavated at the Ortiz site (Fig 3) are interpreted, following the system of Knüsel and Robb, as “Primary Depositions” [39:Table 1]. Even small anatomical articulations were maintained in the Ortiz burials, such as among bones of the hands and feet. This suggests these individuals died in the vicinity of the site and were buried not long after death.

The 1993 excavation’s notes and photographs provide documentation concerning the burial treatments afforded the Ortiz individuals. All five depositions were made in supine, extended position. The positions and orientations of these burials are consistent with burials at the Maruca Site [31]. Burials 1, 2, 3, and 4 were interred in side-by-side alignment near to one another and at the same depth below ground surface (Fig 3). The heads of Burials 1–4 were oriented towards the west; poor preservation of the cranial remains does not allow determining the orientation direction of these individuals’ faces. Excavation field notes record that these four depositions were made into the shell midden matrix and the backfill overlying them formed discrete “pockets” (as if deposited through the dumping of tray or basket loads of shell). Analysis of shells from both midden and burial backfill found no significant differences in the two contexts’ species composition [2]. Excavation notes make no mention of grave cuts or grave walls, and no such features are noted in either plan or profile for B1–B4. The in situ remains for Burial 2 include the legs and feet only. Skeletal elements superior to the legs were missing due to a pit having been excavated there sometime after interment (Fig 3).

The contextual data for B1-B4 suggested to the site’s excavators that they were contemporaneous, or nearly so. Burial 5, on the other hand, is oriented with its head toward the west and the remains show evidence of heating or burning (described below). This indicates its interment was a different event from the burial of the other four individuals. While the burials’ archaeological context indicates that at least two separate burial events took place, the radiocarbon results (presented below) indicate the burials were the results of a series of separate inhumations made over a prolonged period. These interments thus also can be considered a “Collective Deposition,” following Knüsel and Robb [39:Table 1]. The burials’ positioning and proximity suggest that they were marked in some way or that memory of their position was maintained through the centuries.

All five skeletons have significant postmortem damage and modification consistent with weathering Stages 3–5 of Behrensmeyer [115]. This is not surprising given the (sub)tropical climate of southwestern Puerto Rico [37]. Some variability among the burials in taphonomic condition was evident. Burial 3 (B3) was the best preserved of the four individuals. The others, in order of decreasing preservation, were Burial 4 (B4), Burial 1 (B1), Burial 2 (B2), and Burial 5 (B5). The condition of the bones correlates inversely with their radiocarbon dating (see below), an observation for which we do not, at present, have sufficient explanation.

The deteriorated skeletal elements are highly fragmented, with extensive longitudinal and transverse cracking. This has resulted in complete obliteration of most portions of individual bones, in particular any areas of lesser density, such as long bone epiphyses or cranio-facial elements. Remaining materials largely consist of eroded and fragmented long bone diaphyses and certain denser regions of flat or irregular bones (e.g., patella, petrous portion of temporals, mandibular corpus). Bone coloration generally resembled 5YR 3/2 (dark reddish brown), although Burial 5 exhibited some darker/black areas and density changes consistent with heating or burning. Teeth are in generally superior condition to bone, although tooth roots and crowns were degraded and friable in some instances. The combined post-interment taphonomic processes affected both preservation and completeness.

Details on the positioning, treatment, and furnishing of each individual deposition are provided in S1 File.

### Osteology

In terms of basic bone inventory, the five recovered burials averaged ∼20% skeletal completeness. None had fully intact skeletal elements. On an individual basis, B4 was more complete (30%) than the other four, with B1 and B3 being 25% complete, B5 being 15%, and B2 being only 10% complete. Dentition was better represented (except in the case of B2, which had no cranial remains), with an average of nearly twelve identifiable tooth crowns present on a per individual basis for the four burials that had teeth. The dentition recovered ranged from B5, which had one identifiable crown, to B4, from which 30 teeth were recovered. In all these cases, the crowns present were quite friable and the tooth roots even more so.

Only limited cranial and postcranial metric data collection was possible due to fragmentation of the remains. No metric data could be recovered from B2. Data collected for the other four burials are presented in Table 2. In some limited instances, long bone preservation permitted the collection of sufficient antero-posterior and medio-lateral diameter measurements for the calculation of cross-sectional bone geometry. These data are presented in Table 3.

**Table 2:** Metric data collected from Ortiz skeletal remains (all measurements in mm).

Metric (L, R)	B1	B3	B4	B5
Mastoid Length	23.0, x			
Chin Height		34.7	35.1	
Height of Mandibular Body	29.5	39.4	34.4, x	
Breadth of Mandibular Body	10.5		11.6, x	
Minimum Ramus Breadth		x, 36.3	x, 39.0	
Maximum Ramus Breadth			x, 44.4	
Maximum Ramus Height			x, 69.4	
Scapula: Height of Glenoid				x, 39.8
Scapula: Breadth of Glenoid		x, 24.4		x, 26.2
Clavicle: Midshaft AP Diameter	10.8, 10.3	x, 13.0		
Clavicle: Midshaft SI Diameter	8.4, 8.8	x, 11.7		
Clavicle: Circumference at Midshaft	30.3, 30.0	x, 41.0		
Humerus: Min. shaft circumference	47.3, 45.2			
Humerus: Midshaft circumference	47.3, 45.2			x, 68.0
Humerus: Max. midshaft diameter	18.2, 16.2		20.2, x	x, 20.3
Humerus: Min. midshaft diameter	12.1, 12.6		18.7, x	x, 13.4
Radius: Midshaft AP Diameter		12.6, x	10.7, x	
Radius: Midshaft ML Diameter		14.6, x	14.1, x	
Radius: Midshaft Circumference		43.0, x	42.0, x	
Ulna: Midshaft Circumference		44.0, x	45.0, x	
Ulna: AP Diameter		12.3, x	13.7, x	
Ulna: Transverse Shaft (ML) Diameter		14.4, x	11.7, x	
Femur: Max. Midshaft AP Diameter	22.9, 24.2	29.8, 27.7	26.8, 28.9	x, 25.8
Femur: Max. Midshaft ML Diameter	24.1, 22.4	24.9, 26.3	25.1, 24.8	x, 22.2
Femur: Max. Subtrochanteric AP Diameter	27.2, x		23.1, 23.8	
Femur: Max. Subtrochanteric ML Diameter	23.1, x		29.2, 28.9	
Femur: Midshaft Circumference	73.0, 73.4	89.0, 87.0	84.0, 83.0	x, 78.0
Tibia: AP Diameter at Nutrient Foramen		34.0, 32.5	28.8, 31.1	31.0, x
Tibia: ML Diameter at Nutrient Foramen		23.1, 23.1	22.1, 22.3	24.4, x
Tibia: Circumference at Nutrient Foramen		92.0, 89.0	83.0, 87.0	86.0, x

**Table 3:** Cross-sectional bone geometry (CSBG) (antero-posterior (AP)/ medio-lateral (ML) diameter) by skeletal element and side.

CSBG (Left, Right)	B1	B3	B4	B5
Humerus	1.5, 1.3		1.1, x	x, 1.5
Radius		0.9, x	0.8, x	
Ulna		0.9, x	1.2, x	
Femur (subtrochanteric)		1.2, x	0.8, 0.8	
Femur (midshaft)	1.0, 1.1	1.2, 1.1	1.1, 1.2	x, 1.2
Tibia		1.5, 1.4	1.3, 1.4	1.3, x

Tentative determination of biological sex was possible for only four of the five individuals (Table 4). For these, given the poor state of preservation, biological sex should be considered as contingent. Based on limited cranial morphology, Burial 1 was determined to be a probable female, while Burials 3, 4, and 5 were deemed to be probable males. No sex determination could be made for Burial 2.

**Table 4:** Biological sex, age, and stature of Ortiz individuals.

Burial	Sex	Age (years)	Stature (cm)
B1	Female?	45+	130
B2	?	Adult	138
B3	Male?	25–45	149
B4	Male?	25–45	167
B5	Male?	17–25	135

Due to both the condition of the bones and the limited precision of adult age determination, only broad age-at-death ranges could be determined (Table 4). Age assessments were based primarily on assessment of dental development, wear, and attrition. As with the Maruca assemblage [31], all recovered individuals were adults. Burial 1 was determined to be an older adult (45 years or older). Burial 2, being in poorest condition of the five, could only be classified as an adult. Burials 3 and 4 were middle or prime adults (25–45 years). Burial 5 was assessed to be a young adult (17–25 years).

Stature for these individuals was estimated based on *in situ* measurements recorded by the excavators (Table 4). The state of osteological preservation did not allow for extrapolation of living stature from long bone lengths. The stature results of the Ortiz individuals ranged from 130 cm to 167 cm. This range slightly exceeds that observed at Maruca [31]. Stature for the three possible males ranged from 135–167 cm and was 130 cm for the sole possible female.

As with the other categories of osteological data, observations of pathology and trauma were limited by the conditions of preservation. The only noteworthy “pathological” condition observed was the extreme state of occlusal dental wear in the teeth of Burial 1, which resulted in near obliteration of dental crowns. This was the case with both anterior and cheek teeth, attesting to possible masticatory and non-masticatory sources of wear. Wear was so extreme that it progressed to the root bifurcation in some instances, particularly on the buccal aspect of first molars.

### Radiocarbon dating

**Fig 4:**
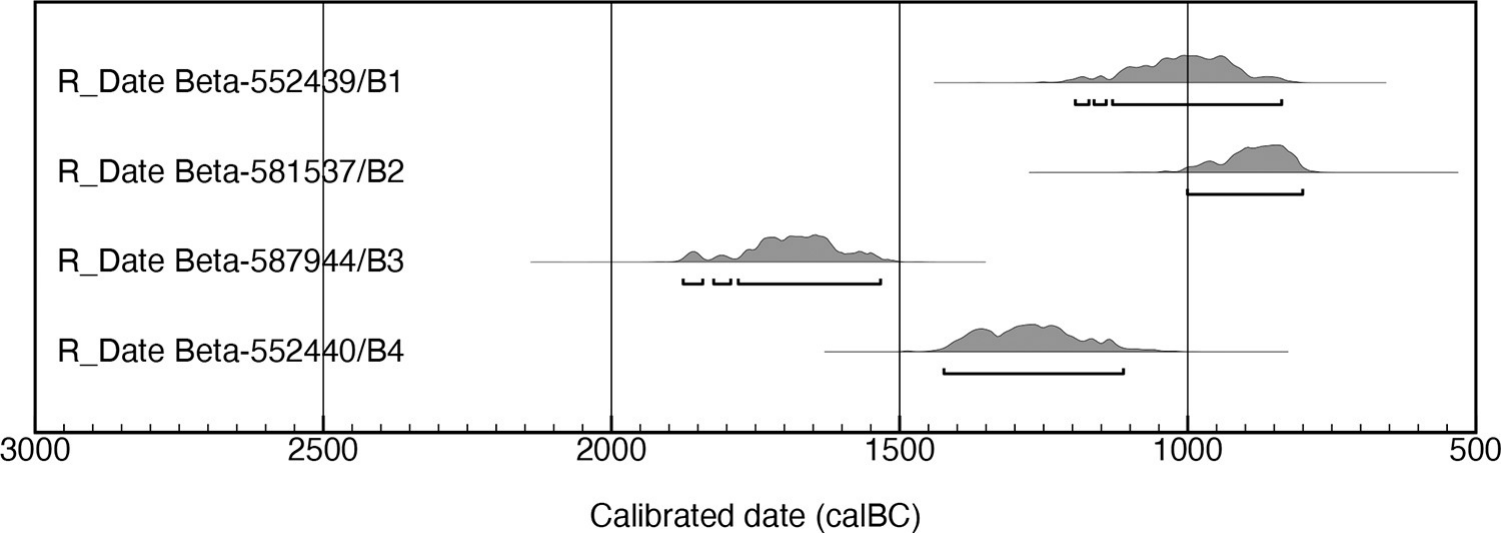
Calibrated ranges of Ortiz burial dates.

Both uncalibrated and calibrated radiocarbon dates for B1, B2, B3, and B4 are presented in Table 5, with calibrated ranges illustrated in Fig 4.

**Table 5:** Radiocarbon (AMS) dates of Ortiz burial bone collagen with calibration parameters and 2-sigma calibrated ranges.

Sample	Burial	Uncalibrated date (rcybp)	*δ*13C (‰)	% Marine used in calibration	Range (calBC)	%
Beta-552439					1200–1170	2.2
	B1	2920±30	-14.9	25.5±14.9	1160–1140	2.2
					1130–840	91.0
Beta-581537	B2	2800±30	-15.5	21.4±12.6	1000–800	95.4
Beta-587944					1880–1840	5.5
	B3	3460±30	-15.7	21.0±13.6	1820–1790	3.5
					1780–1530	86.4
Beta-552440	B4	3110±30	-15.1	25.1±15.4	1420–1110	95.4

Based on these results, the Ortiz burials are, at present, the oldest directly dated human burials from the island of Puerto Rico. Reniel Rodríguez Ramos’ exhaustive database of radiocarbon assays from both published sources and unpublished CRM reports includes some 243 other human bone dates from sites across the island [116]. Of these, only three have uncalibrated radiocarbon dates older than 1600 rcybp. Two of these are from the Maisabel Site [117] and one from Site AR-39 [118].

It is difficult to gauge exactly how much older the Ortiz remains are than others from the island, given that we cannot fully account for marine reservoir effects in calibration of previously dated human remains from other sites. Even the most recent date from the Ortiz burials, Burial 2, with uncalibrated age 2800±30 bp, predates the island’s next oldest directly dated human burials by roughly 1200 uncalibrated radiocarbon years. While older dates are available from other early period sites, none of them are direct dates obtained from human bone.

In terms of the Ortiz Site radiocarbon date sequence, Burial 3 appears to be significantly earlier than any of the other recovered burials (1880–1530 calBC). Its 2-sigma range is exclusive of any of the other burials. There is a minimum one-century gap between it and the next interment’s radiocarbon date, Burial 4 (1420–1110 calBC). The calibrated range of Burial 4 overlaps the 2-sigma range of Burial 1 (1200–840 calBC). Burial 2 has the most recent radiocarbon date (1000–800 calBC). Thus, the apparent overall sequence of these four burials from earliest to latest is as follows: Burial 3, followed by a gap of at least a century, then Burial 4 being possibly contemporaneous with Burial 1, and finally Burial 2 being possibly contemporaneous with Burial 1. The order of B4/B1 and B1/B2 cannot be stated with certainty.

### Paleodietary/Stable isotope analysis

Data on chemical and elemental preservation of the Ortiz burials is found in Table 6. While collagen yield exceeded the 0.5 wt% minimum value for isotope analysis set by Hedges and van Klinken [119] and van Klinken [120], none of the five samples yielded more than the 3.5 wt% collagen preferred value established by Ambrose [121]. Given the climatic conditions of southwestern Puerto Rico and the radiometric age of these samples, this is not surprising [37]. Notwithstanding these low chemical yields, the carbon and nitrogen elemental composition of B1–B4 exceeded the lower cutoff values for *bona fide* collagen offered by Ambrose [121], ranging from 6.9–40.8 wt% carbon and 2.3–14.0 wt% nitrogen respectively. (B5 did not meet this standard.) The atomic C:N ratios of B1–B4 all fell within the preferred 2.9–3.6 range [121]. Hydroxyapatite yields for the four samples are 40.5–64.6 wt% and fall within or slightly above the 21–63 wt% range recently offered by Chesson and colleagues [122] as bounding well-preserved bone hydroxyapatite.

**Table 6:** Chemical, elemental, and isotopic data for Ortiz and Maruca individuals.

Burial	Collagen yield	wt% C	wt% N	Atomic C:N	*δ*^13^C (‰)	*δ*^15^N (‰)	Apatite yield	*δ*^13^C_ap_ (‰)	*δ*^18^O_ap_ (‰)
B1	2.6%	31.6%	10.2%	3.6	-14.9	9.5	43.6%	-6.3	-1.7
B2	3.2%	39.7%	13.6%	3.4	-15.5	9.4	64.6%	-8.4	-1.5
B3	2.7%	6.9%	2.3%	3.5	-15.7	7.2	63.3%	-6.4	-2.2
B4	3.2%	40.8%	14.0%	3.4	-15.1	9.3	40.5%	-5.7	-2.0
B5	1.7%	-	-	-	-	-	-	-	-
Maruca 2B	1.3%	30.2%	10.2%	3.5	-18.3	8.9	-	-8.8	-

Measured stable isotope ratios for the four sufficiently well-preserved Ortiz individuals are presented in Table 6. *δ*^13^C_co_ for these four individuals averaged -15.3±0.4‰, *δ*^15^N_co_ 8.9±1.1‰, and *δ*^13^C_ap_ -6.7±1.2‰. Values for both carbon isotope systems are notably ^13^C enriched in the Ortiz samples compared with those observed for other early period individuals from Maruca (-18.3‰ for *δ*^13^C_co_ (n = 1) and -11.7±2.8‰ (n = 3) for *δ*^13^C_ap_). *δ*^15^N_co_ values are similar to the one Maruca individual (8.9‰) for whom there is nitrogen stable isotope data [32:Table 11.2].

*δ*^13^C_co_ and *δ*^13^C_ap_ values for the Ortiz site individuals are also higher than the average *δ*^13^C_co_ -18.1±1.0‰ and -8.9±1.2‰ *δ*^13^C_ap_ observed for the 229 Puerto Rican Ceramic Age individuals. Conversely, nitrogen stable isotope values of the Ortiz individuals are slightly lower than those observed for the Ceramic Age individuals (9.7±0.8‰).

Results of FRUITS modeling for B1–B4 are presented in Table 7. Freshwater/terrestrial fauna (FWTERR) make up the single largest modeled dietary contributor for B1, B3, and B4, ranging from 30.9% to 33.9% of total calories consumed. Burial 2 shows a reversed relationship, with C_3_ plants making up 37.5% of diet versus 26.8% for FWTERR. For all four individuals, the contribution of protein rich FWTERR terrestrial fauna is greater than the proportion of the marine food groups (M1 and M2) combined. Marine 1 (higher trophic level marine animals) makes up 7.8–12.1%, while Marine 2 (reef and seagrass fauna) contributed 9.4–13.7%. Combined, M1 and M2 averages 23.3±2.4% of individual diet. The ratio of terrestrial to marine protein sources varies from 1.2:1 to 1.6:1 among the Ortiz individuals. This finding is in stark contrast to the impression of dietary protein given by analysis of faunal remains from the site’s midden, an assemblage which consists overwhelmingly of marine invertebrate remains [2]. Considering plant foods for all four individuals, C_3_ plants make up a larger portion of diet, averaging 28.7±6.1%, than C_4_/CAM plants, which averaged 16.8±1.7%. Individual C_3_:C_4_/CAM ratios range from 1.3:1–2.6:1.

**Table 7:** Results of FRUITS paleodietary modeling for Ortiz and Maruca individuals.

Burial	C_3_ mean	sd	C_4_/CAM mean	sd	FWTERR mean	sd	M1 mean	sd	M2 mean	sd	Total marine mean	sd
B1	26.4%	14.7%	17.2%	12.5%	30.9%	20.1%	11.8%	9.8%	13.7%	11.2%	25.5%	14.9%
B2	37.5%	16.1%	14.3%	10.4%	26.8%	20.9%	12.1%	9.7%	9.4%	8.1%	21.4%	12.6%
B3	27.7%	15.9%	17.4%	12.2%	33.9%	21.8%	7.8%	7.6%	13.3%	11.2%	21.0%	13.6%
B4	23.3%	14.3%	18.3%	13.1%	33.4%	20.3%	11.1%	9.7%	14.0%	12.0%	25.1%	15.4%
*Ortiz (avg*.*)*	*28*.*7%*	*6*.*1%*	*16*.*8%*	*1*.*7%*	*31*.*2%*	*3*.*2%*	*10*.*7%*	*2*.*0%*	*12*.*6%*	*2*.*2%*	*23*.*3%*	*2*.*4%*
Maruca 2B	43.5%	19.4%	12.0%	9.0%	28.3%	21.8%	7.7%	6.8%	8.5%	7.3%	16.2%	10.0%

**Fig 5:**
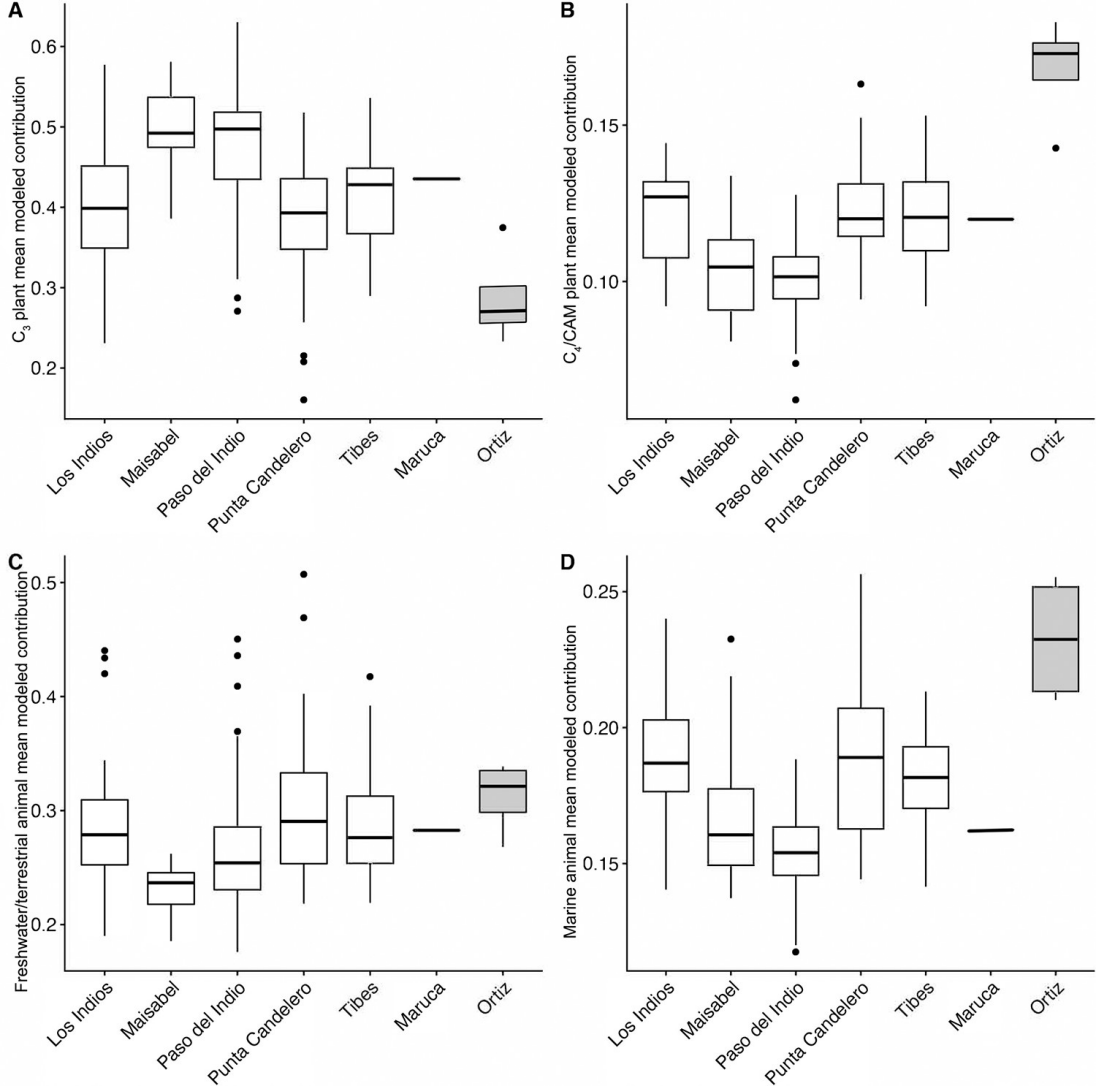
Quadplot comparing results of FRUITS paleodietary modeling for Ortiz, Maruca, and Ceramic Age individuals.

Compared with Maruca 2B, the only other early period individual for whom we possess modeled paleodiet (Table 7), the Ortiz individuals had a diet composed of roughly ten percent more animal food if FWTERR, M1, and M2 contributions are combined (54.5% vs. 44.5%). The Ortiz inhabitants also consumed a somewhat greater quantity of C_4_/CAM plants (16.8% vs. 12.0%) and a far lesser quantity of C_3_ plants (28.7% vs. 43.5%). Similar relationships hold when the Ortiz individuals’ diet is compared with the modeled diet of the 229 Ceramic Age individuals. The four Ortiz individuals consumed more freshwater and terrestrial fauna (31.2% vs 27.8%), more high trophic level marine fauna (10.7% vs. 9.1%), more reef and seagrass marine fauna (12.6% vs. 8.2%), more C_4_/CAM plants (16.8% vs. 11.3%), and far fewer C_3_ plants (28.7% vs. 43.6%) than most of their Ceramic Age counterparts (Fig 5).

**Fig 6:**
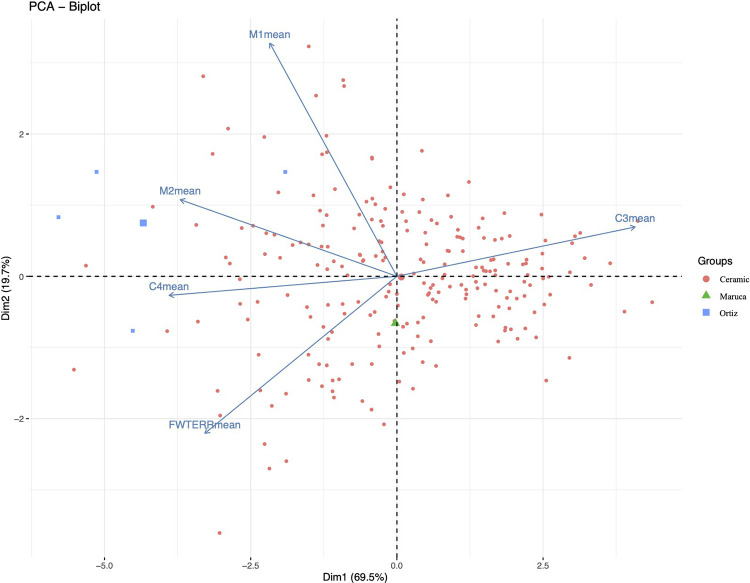
PCA plot of FRUITS paleodietary modeling of Ortiz, Maruca, and Ceramic Age individuals.

The overall dissimilarity of the Ortiz individuals’ diet from that of both the Maruca individual and the vast majority of the Ceramic Age individuals is best reflected in the PCA plot displayed as Fig 6. The positioning of the Ortiz individuals is notably distinct while not exclusive of the Ceramic Age comparanda but falls far from the only other early individual from Maruca.

### Sr isotopes

Results of strontium isotope analysis for B1, B3, B4, and B5 are presented in Table 8. While the range of measured individual ^87^Sr/^86^Sr signatures is only 0.0006, these results nonetheless indicate potentially meaningful differences among the four analyzed individuals’ places of childhood residence.

**Table 8:** Strontium isotope (^87^Sr/^86^Sr) analysis of Ortiz individual dentition.

Burial	Sr Tooth	^87^Sr/^86^Sr	95% CI
B1	Left mandibular M2	0.70816	0.00003
B2	-	-	-
B3	Right mandibular M1	0.70803	0.00005
B4	Left maxillary M1	0.70797	0.00006
B5	Lower M	0.70756	0.00004

**Fig 7:**
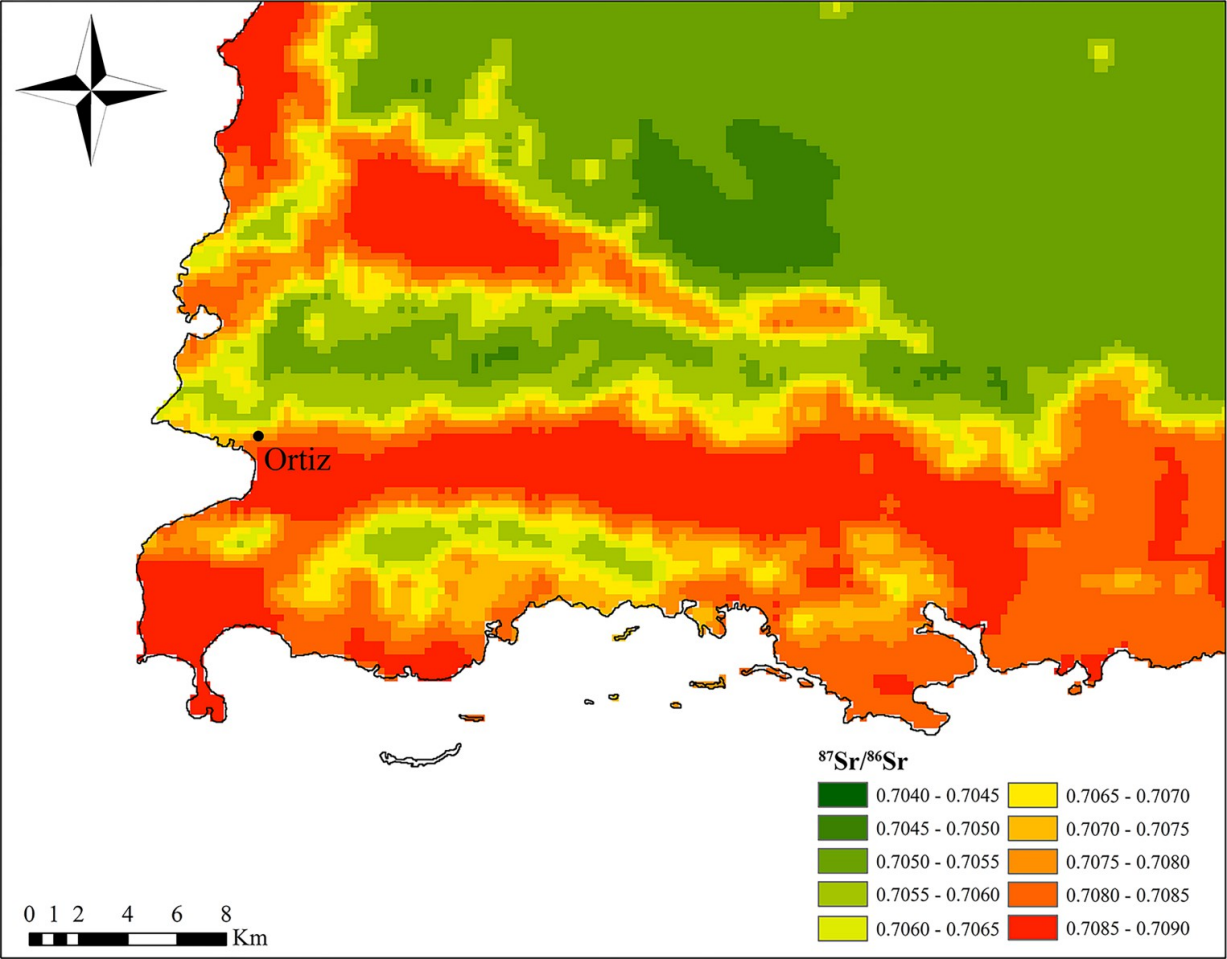
Strontium (^87^Sr/^86^Sr) isoscape of southwestern Puerto Rico.

Compared to the range of local ^87^Sr/^86^Sr signatures (Fig 7), which vary significantly due to local geological heterogeneity, all four measured individuals could be of “local” origin (within kilometers/tens of kilometers of the Ortiz site), but the variance among them may speak to subtle differences in place of early life residence and/or varied patterns of early life mobility. If all four had been born or raised exactly at the Ortiz site itself, we would expect less variation in their ^87^Sr/^86^Sr signatures than was observed.

## Discussion and conclusion

The overarching goal of the present study was the elucidation of the lived experiences of the five Ortiz site individuals. While such reconstructions necessarily are highly dependent upon factors such as preservation and contextual control, neither of which is exemplary in the present instance, the multidisciplinary approach employed nonetheless yielded important insights into the lives of these five early Puerto Rican inhabitants. We consider in turn the seven specific research questions that guided our analyses.

### Funerary traditions

Considering the Ortiz burials in comparison with the burials from Maruca, the island’s only other large and well-studied early skeletal assemblage [31], we find a generally consistent canon of mortuary practices. As with the eleven individuals from Maruca, all five burials from Ortiz were single primary interments in a supine position made directly in the earth (with the possibility of shrouds/other organic wrapping/ties as discussed above). Moreover, all five of the Ortiz burials were buried with their legs in an extended position, which was also the case for ten of the eleven Maruca individuals, and with their arms positioned alongside the body or with their hands positioned atop their trunk/pelvis. Four out of five of the bodies at Ortiz had their heads placed at the west end of the burial and the fifth had its head placed at the north end of the interment. This is consistent with the pattern observed at Maruca where the majority (six of eleven) of the burials had the head oriented west and a further two of eleven had the head oriented from north to northwest. Even the grave good assemblages at Ortiz seem largely similar in composition (shell, lithics, coral, and ochre) to those provided for the Maruca individuals. The pendants provided to all five of the Ortiz burials are the one apparent departure from this common corpus of early grave goods (see below). Finally, it is intriguing to note that the excavators of Maruca also observed what they described as incidental postmortem heat alteration of some skeletal elements, which also may have been the case with Ortiz Burial 5.

In his work on Maruca [31], Crespo-Torres observed that the paucity of data (from islands other than Cuba) on the mortuary practices of the insular Caribbean’s earliest inhabitants made it all the more important to be aware of, and to document, possible regional variations in funerary customs. Taking together, the two demonstrably early southern and southwestern Puerto Rican mortuary sites of Maruca and Ortiz begin to inform an early regional mortuary canon that can be juxtaposed with early practices observed elsewhere. This is admittedly just a first approximation, but the consistencies observed in grave type, body position, orientation, grave goods, and possible postmortem interaction with the dead via the medium of fire at both sites, would seem to suggest a set of shared funerary customs in southern Puerto Rico during the final two millennia BC. It is unfortunate that we do not possess direct dates from the Maruca burials that might enable us to assess whether that assemblage, like Ortiz, represents centuries to millennia of continued and consistent mortuary practices.

### Taphonomy

The general taphonomic process common to all five burials would appear to have consisted of repeated wet/dry cycles resulting in collagen hydrolysis, gross swelling and shrinkage, crack propagation, and ultimate fragmentation or destruction of bone. Similar processes have been observed in many Ceramic Age open-air burials from across Puerto Rico [37], and the greater antiquity of the Ortiz burials (see below) explains their uniformly poor preservation. Further damage was caused by past agricultural plowing which left the articulated remains immediately below the site’s stratigraphic plow zone.

### Biological profile

Osteological analysis of the Ortiz burials determined that all five of the individuals were adults at the time of their deaths. It is unknown whether the absence of non-adults is the result of differentiated mortuary practice, the effects of differential preservation, or simply because non-adults were buried in an unexcavated portion of the site. Sex determination, which was minimally successful for four of the burials, identified three probable males and one probable female. We did not observe any grouping of burials by sex as was seen at Maruca and we do not attribute any significance to the possibly skewed sex distribution of this small sample [31].

### Chronology

These radiocarbon dates obtained from the bone collagen of four of the Ortiz individuals indicate a broad temporal distribution for the site’s burials. It one considers the earliest possible date (1880 calBC) of the 2-sigma range for Burial 3, and the recent end of the 2-sigma calibrated range of Burial 2 (circa 800 calBC), it appears that the Ortiz site may have been consistently or recurrently used as a mortuary space for more than 1,000 years. Even at the most proximate ends of the radiocarbon date calibrated ranges of the site’s earliest and most recent interments (1530 calBC for Burial 3 and 1000 calBC for Burial 2), mortuary activities at the site would have persisted for over 500 years, a span of more than twenty 25-year generations. Some implications of this finding are presented below.

These radiocarbon dates are incongruous with inferences derived from the burials’ archaeological context (side-by-side burial near to one another at the same depth), which suggested that B1–B4 were contemporaneous, or nearly so. While we are generally quite confident that these dates accurately represent the recurrent use of the formal mortuary space for centuries, it should be noted that other phenomena may have affected the integrity of these dates. First, a different collagen extraction protocol was used for Burial 3 versus Burials 1, 2, and 4, which could engender some difference in dating. Second, the differential contribution of different marine food sources (M1 versus M2, which represent different trophic levels and marine habitats) to the different individuals’ diets could introduce source-dependent reservoir effects, thus distorting the radiocarbon dates obtained. Both of these possibilities merit future examination.

### Paleodiet

This similarities between the Maruca and Ortiz individuals does not continue in respect to diet, at least as far as the small number (n = 5) of isotopically analyzed individuals from the two sites would seem to suggest. Stark differences in both carbohydrate and protein consumption at the two sites serve as a reminder that cultural similarities in one regard (mortuary customs) need not oblige uniformity in other domains (diet). Indeed, the lack of perfect concordance suggest that we should be cognizant of the possibility of quasi- or entirely independent variability functioning at multiple scales (insular, regional, site, and individual, to name a few), among and between even putatively contemporary early peoples, rather than assuming cultural homogeneity within any given age/period. That the Ortiz individuals consumed far less C_3_ plants, a greater proportion of C_4_/CAM carbohydrates, and more marine protein than observed at Maruca is clear, even if the reasons why are not evident. Given some of the differences in local climate and biogeography between the two sites, we cannot help but wonder if disparities in locally available resources conditioned the observed differences in patterns of consumption. To be certain, aspects of the diet of the Ortiz individuals resulted in a pronounced degree of dental wear. Whether this wear ought to be termed “pathological” is questionable, as it may instead represent the normal wear trajectory of teeth for this population, given that B1 is the oldest of the recovered and analyzed individuals. Pronounced dental wear of the type documented here was also observed in the early population from Maruca [31].

Comparison of these early inhabitants’ diets and those of later Ceramic Age peoples provides conflicting impressions of the potential cultural dissimilarities between these two groups, a topic of great debate across the island and throughout the archipelago [123–130]. The diets of the four analyzed Ortiz individuals consisted of a far different combination of plant and animal foods than has been found in the hundreds of Puerto Rican Ceramic Age individuals studied to-date, and yet the one Maruca individual had a diet the was generally consistent with the members of these later populations. All caveats of small sample size being acknowledged, we find in these data suggestions of both cultural distinctiveness and similarity between Ceramic Age peoples and different groups of antecedent peoples. If one seeks to distill evidence of transculturation or cultural influence of early peoples on the island’s later arrivals, these data may indicate greater affinities between Ceramic Age peoples and some early groups than others. Connections of differing kinds and intensity with pre-existing early peoples could help explain the emergence of the pronounced cultural variability seen in Puerto Rico from the middle Ceramic Age (ca. AD 500) onwards [116].

### Paleomobility

Sr isotope analysis of four of the Ortiz site burials indicate that, while the individuals likely lived the early portions of their lives in the environs of the site (modern-day Boquerón/Cabo Rojo), they do not all seem to have been born and raised at the site itself. The geological, and thus isotopic, heterogeneity depicted in Fig 7 makes clear that these individuals could have possessed varied Sr isotope signatures while still being “locals”, hailing from settlements within a few/tens of kilometers of the site. It thus appears that the Ortiz site may have served as a common mortuary space for people of different *local* communities, people who were buried in a common locale due to some centripetal cultural force that dictated their burial in such a common or shared place. As we discuss below, this fact, combined with the longevity of the site as a mortuary space, has important social implications.

### Broader dimensions of early Puerto Rican lifeways

Perhaps the most significant findings of the present study derive from radiocarbon dating and Sr isotope analysis, which combine to describe a persistent and formalized mortuary space containing the burials of individuals from different nearby places of early-life residence. This is not to say that the Ortiz Site was unique or remarkable for the early period, for similar conclusions could have been generated from analysis of the Maruca burials, were a detailed chronology and assessment of paleomobility available for those interments. Furthermore, we acknowledge that any broader social conclusions derived from our findings are contingent due to the small sample size involved, even though the Ortiz burials represent 20–25% of the total burials from the island’s early period. Rather, we present the following broader potential implications relating to the Ortiz site precisely because we have generated data that indicate the long-term use of a central and formal mortuary space with internally consistent burial practices spanning 500–1,000 years.

Saxe’s Hypothesis 8 proposed that, “to the degree that corporate group rights to use and/or control crucial but restricted resources are attained and/or legitimized by means of lineal descent from the dead (i.e., lineal claims to ancestors), such groups will maintain formal disposal areas for the exclusive disposal of their dead, and conversely,” [131]. Application of this hypothesis, which was validated by the work of Goldstein [132], to the Ortiz site, raises the possibility of the existence in the early period of southwestern Puerto Rico of corporate groups making territorial/resource claims via their use of a formal mortuary space. What resources were being laid claim to remains to be demonstrated but could include nearby shellfish beds and/or sources of lithic raw material. The persistent use of a common canon of mortuary treatment for centuries can be seen as further evidence of assertions of common group identity [133], which is even more intriguing given that the Ortiz individuals would seem to hail from different settings within the local region. The particular significance of the pendants found with the Ortiz burials is a topic of present ongoing study. They may have served as a particularly salient and visible means of linking together the individuals buried at Ortiz and tying them to that particular “place” [134].

In reviewing correlates of Hunter-Gatherer mortuary variability, Hofman first established that the recurrent use of formal cemetery spaces often is linked with more consistent and predictable resource availability, and thus reduced group mobility [135], typifying logistical, rather than residential, mobility of Binford [136]. Furthermore, Hofman found that the proportion of primary burials found at a mortuary site further indicates decreased residential mobility, as secondary burials are required when mobile groups return deceased individuals to a formal and central place of burial postmortem [135]. In light of this, the Ortiz interments may be evidence for: 1) the existence of corporate groups with shared identity making territorial claims through the use of formal mortuary spaces and common burial practices, and 2) that this group engaged in a limited degree of mobility, finding all that they needed (economically and socially) to sustain life in the immediate environs of Boquerón Bay and Cabo Rojo. The site’s persistence and the long-term maintenance of cultural (mortuary) practices further problematizes long-held characterizations of the island’s earliest inhabitants as itinerant food foragers constantly on the move in search of the necessities of life.

Finally, the data presented here may serve to identify the Ortiz site as a persistent place in the landscape of southwestern Puerto Rico. “Place” is defined as a location of importance, meaning, and connection in human/cultural geography, while “persistent places” are defined in anthropology/archaeology as locations to which groups continuously return for certain activities [137]. Persistent places have received a great deal of attention in archaeology over the past several decades, fomenting robust discussion on their relationship to a variety of other central cultural, social, and political phenomena [138–143]. The phenomenon of site persistence and place-making that appears to characterize Ortiz has been observed previously at sites from across the island, including Maruca, Paso del Indio, and Puerto Ferro [125]. This notion of place-making forms part of a growing body of literature from Puerto Rico, as well as the Antilles more broadly, which documents anthropogenic modification of the Caribbean islandscapes by their first inhabitants [144–149]. Such a finding adds further credence to evidence that, “indicates that rather than their spatiality being driven by the distribution of biotic resources as is often assumed in hunter-gatherer societies, these people were capable of manipulating the environment and transforming it into an artifact of their own making,” [125]. This realization recognizes the key role that a landscape’s physiographic character plays in conditioning human settlement patterning, land use, and cultural adaptation over long spans of time, as demonstrated in Koski-Karell’s wide-ranging study of pre-Columbian settlement patterns in northern Haiti [150], while still acknowledging the active agency of these prehistoric peoples in shaping the landscapes they inhabited. While such an attribution of agency need not equate to higher levels of social “complexity” for these early societies [151], it does suggest that through their active use, and re-use, of certain spaces, these societies were making persistent places that, in turn, structured later activities, and contributed to binding and persistent social memory, thereby building a, “landscape of meaning,” [152].

In the Ortiz burials, then, we find not just evidence of the individual lifeways of five of the earliest documented inhabitants of Puerto Rico, but tantalizing indications of the existence of a mode of life on the island’s southwestern corner that defies many earlier characterizations of the “Archaic”. While many of the ideas developed here are in their early stages and are the subject of ongoing work in the region, they lend further credence to a growing realization that the lives of the earliest Puerto Ricans may have occurred in a social and cultural context that was rather more complex than we have long assumed. The apparent presence, in this one region at least, of persistent places possibly used for centuries by corporate groups held together by a shared identity and sustained by a means of subsistence that required only limited logistical mobility, raises the potential for the discovery of a far more vibrant and varied cultural landscape in the earliest period of Puerto Rico’s inhabitation. These findings offer a challenge to archaeologists working across the island to develop new and better understandings of these earliest lifeways in hopes of painting a more accurate and nuanced picture of the lives of the first Puerto Ricans.

## Supporting information

S1 File Ortiz burial contextual data. Document provides detailed contextual data for each of the five Ortiz burials.
(DOCX)
