# Supplementary material for: Reconsidering the lives of the earliest Puerto Ricans: Mortuary Archaeology and bioarchaeology of the Ortiz site
Source: PLoS One. 2023 Apr 26;18(4):e0284291. doi: 10.1371/journal.pone.0284291 (PMC10132640; doi:10.1371/journal.pone.0284291)
Supplement: S1 File — Document provides detailed contextual data for each of the five Ortiz burials. (DOCX) [file pone.0284291.s001.docx]

**Burial 1**

The second burial from the north in the row of four east–west oriented depositions. It lies south of Burial 2 and north of Burial 3 (Fig. 3). Skeletal elements of this burial were found between 15 and 30 cm below surface. B1 was supine and extended with the individual’s head positioned to the west. The individual’s right arm and hand were supinated and positioned atop the pelvis. Excavation notes and photographs record that the left arm and hand were pronated and positioned below the left hip bone with the ulna atop the radius. The arm was twisted so that hand rotated laterally with thumb downwards, an awkward anatomical position. B1’s legs were noted by the excavators as being very close to one another and possibly tied together. There is no direct evidence supporting this interpretation, although suggestion of similar limb binding has been noted for certain of the Maruca burials (31). Objects associated with this deposition include a group of rocks (“cairn”) in the pelvic area that included grinding or ground stones and ochre. There were also several lithic blades, shell artifacts (scrapers and pointed “tips”), a stone amulet near the abdomen, and a “stone peg” near the left foot. Both the amulet and the “peg” are the subject of ongoing analysis. Peg-shaped stone artifacts are associated with the early inhabitants of Hispaniola, west of Puerto Rico (1).

**Burial 2**

The northernmost of the four east–west oriented depositions, lying approximately 40 cm north of Burial 1 (Fig. 3). Skeletal elements of this burial were found between 14 and 28 cm below surface. Burial 2 appears to have been supine and extended, with head positioned to the west/northwest. The direction is inferred from the orientation of the lower limbs and some skull fragments, which are all of B2 that remained undisturbed. This deposition was dramatically affected by the later digging of a pit, plowing action, or other disturbance. Objects associated with this deposition include chert cobbles, scrapers, and blades, a piece of red ochre, a hammer stone, and a coral piece that was smoothed as if used as a file.

**Burial 3**

The third burial from the north in the row of four east–west oriented depositions. The scale in Fig. 3 shows that it lies 25 cm south of Burial 1 and 18 cm north of Burial 4. Skeletal elements of this burial were found between 17 and 30 cm below surface. B3 was supine and extended with head positioned to the west. The individual’s right arm and hand were supinated and positioned parallel to the thigh. The left hand was supinated and positioned atop the pelvis. Objects associated with this deposition include a group of five rocks on top of pelvis, several lithic blades, a hammer stone, a piece of red ochre, and an incised serpentine amulet or pendant found in the area of the abdomen. This amulet is currently under study.

**Burial 4**

The southernmost in the row of four east–west oriented depositions, lying 18 cm south of B3. This burial’s skeletal elements were found between 20 and 30 cm below the surface. B4 was supine and extended with head positioned to the west. This individual’s face may have been oriented facing south. The left hand was supinated and positioned next to or on top of the pelvis-left thigh area. The right hand was supinated and positioned alongside the right hip bone. Objects associated with this deposition include several lithic blades, scrapers, and flakes along with shattered chert fragments, a coral piece smoothed as if used as a file, and several intact shells. There was part of a broken conical *mano* grinding stone in the area of the skull.

**Burial 5**

This deposition was positioned west of Burials 1-4 (Fig. 3). Its skeletal elements were found between 18 and 28 cm below surface. B5 was supine and extended with head positioned towards the north (unlike the other four burials). The individual’s right arm and hand were supinated and positioned on top of the right side of the pelvis. The left hand was supinated and positioned alongside the left thigh. Burial 5 showed evidence of partial burning, which the excavators interpreted as resulting from possible “low heat” cremation fire. Evidence of B5’s partial heating or burning is apparent on bones of the skull, right arm, right ribs, left femur, and right tibia. Other bones including the left humerus, right femur, and right tibia, show no such heat modification. The apparent anatomical articulation and positioning of the bones making up B5, including small bones of the hands and feet, and the uneven burning or charring of various skeletal elements, indicate that full immolation of the remains did not occur prior to the grave being backfilled. An alternative to the body being affected by funerary or other intentional burning is that the B5 internment was unintentionally exposed to heat or flame following primary inhumation, perhaps long after. Similar burned bone observed at the Maruca Site burials was interpreted as resulting from postmortem or post-depositional incidental exposure to heat or fire (31). The circumstances regarding B5’s heating or burning remain unclear. Objects associated with this deposition include a group of three rocks close to the pelvis. There were also several lithic blades, a piece of red ochre, and a possibly burnt amulet. Excavation notes record that shell was found only in the burial’s chest and head area, not with or near the individual’s legs.

At least one stone pendant/pendant fragment was found with each of the five burials. Three of the five burials (B1, B3, and B5) were found to have rocks directly on top or alongside the pelvis but lacked coral pieces. Conversely, Burials 2 and 4 had one associated coral piece each, along with various lithic tool implements. These coral pieces appear to have been used for filing. They are cylindrical, worn nearly smooth as if from use as a file, and are of length and diameter easily held in one’s hand. B2 and B4 did not contain rocks on or near the pelvis, nor an amulet. These two groups of burial-associated artifacts may indicate purposeful deposit of different grave goods based on sex or some other factor, although the osteological analysis (discussed below) suggests the grave goods may not correlate with the sex of the burials.
